# Macro- and Micronutrients Consumption and the Risk for Colorectal Cancer among Jordanians

**DOI:** 10.3390/nu7031769

**Published:** 2015-03-10

**Authors:** Reema F. Tayyem, Hiba A. Bawadi, Ihab N. Shehadah, Suhad S. Abu-Mweis, Lana M. Agraib, Kamal E. Bani-Hani, Tareq Al-Jaberi, Majed Al-Nusairr, Dennis D. Heath

**Affiliations:** 1 Department of Clinical Nutrition & Dietetic, The Hashemite University, P.O. Box 150459, Zarqa 13115, Jordan; E-Mails: suhad.abumweis@hu.edu.jo (S.S.A.-M.); elonafrsh2003@yahoo.com (L.M.A.); 2 Department of Health Sciences, College of Arts and Sciences, Qatar University, P.O. Box 2713, Doha, Qatar; E-Mail: hbawadi@qu.edu.qa; 3 Chief Gastroenterology Division, King Hussein Cancer Center, P.O. Box 35102, Amman 11180, Jordan; E-Mail: ishehadeh@khcc.jo; 4 Faculty of Medicine, The Hashemite University, P.O. Box 150459, Zarqa 13115, Jordan; E-Mail: k_banihani@hu.edu.jo; 5 Department of General and Pediatric Surgery, Jordan University of Science and Technology, P.O. Box 3030, Irbid 22110, Jordan; E-Mail: tmrjaberi@hotmail.com; 6 Chief Gastroenterology Division, Prince Hamza Hospital, P.O. Box 86, Amman 11118, Jordan; E-Mail: jwan97@hotmail.com; 7 Cancer Prevention and Control Program, Moores Cancer Center, University of California, San Diego, La Jolla, CA 92093, USA; E-Mail: dheath@ucsd.edu

**Keywords:** colorectal cancer, total energy, macronutrient, micronutrients

## Abstract

Objective: Diet and lifestyle have been reported to be important risk factors for the development of colorectal cancer (CRC). However, the association between total energy and nutrient intake and the risk of developing CRC has not been clearly explained. The aim of our study is to examine the relationship between total energy intake and other nutrients and the development of CRC in the Jordanian population. Research Methods and Procedures: Dietary data was collected from 169 subjects who were previously diagnosed with CRC, and 248 control subjects (matched by age, gender, occupation and marital status). These control subjects were healthy and disease free. Data was collected between January 2010 and December 2012, using interview-based questionnaires. Logistic regression was used to evaluate the association between quartiles of total energy, macro- and micronutrient intakes with the risk of developing CRC in our study population. Results: Total energy intake was associated with a higher risk of developing CRC (OR = 2.60 for the highest *versus* lowest quartile of intake; 95% CI: 1.21–5.56, *p*-trend = 0.03). Intakes of protein (OR = 3.62, 95% CI: 1.63–8.05, *p*-trend = 0.002), carbohydrates (OR = 1.41, 95% CI: 0.67–2.99, *p*-trend = 0.043), and percentage of energy from fat (OR = 2.10, 95% CI: 0.38–11.70, *p*-trend = 0.009) significantly increased the risk for the development of CRC. Saturated fat, dietary cholesterol and sodium intake showed a significant association with the risk of developing CRC (OR = 5.23, 95% CI: 2.33–11.76; OR = 2.48, 95% CI: 1.18–5.21; and OR = 3.42, 95% CI: 1.59–7.38, respectively), while vitamin E and caffeine intake were indicative of a protective effect against the development of CRC, OR = 0.002 (95% CI: 0.0003–0.011) and 0.023 (95%CI: 0.008–0.067), respectively. Conclusion: Our results suggest an increased risk for the development of CRC in subjects with high dietary intake of energy, protein, saturated fat, cholesterol, and sodium, and diets high in vitamin E and caffeine were suggestive of a protective effect against the risk of developing CRC. Impact: This is the first study in Jordan to suggest that it may be possible to reduce CRC risk by adjusting the intake of some macro-and micronutrients.

## 1. Introduction

Published report suggests that colorectal cancer (CRC) is one of the three most common forms of cancer with nearly 1.4 million new cases diagnosed in the year 2012 [1]. The report noted that the Republic of Korea, followed by Slovakia and Hungary had the highest incidence of diagnosed CRC, while the lowest incidence of diagnosed CRC was in Africa and Asia [1]. The National Cancer Registry of Jordan reported 554 diagnosed cases of CRC in the year 2009. This total number of cases was calculated to be 11.9% of all newly diagnosed cancer cases in the kingdom of Jordan [2]. Cancer is described in part by an abnormal cell growth that is believed to be initiated either by internal or external (environmental) factors [3]. Diet and physical activity are external factors that may play a role in the development of CRC disease [3].

Dietary intakes of energy, macro- and micro-nutrients have been implicated in the etiology of CRC [4]. Several studies have shown that high dietary intakes of energy and energy-supplying macronutrients (fat, protein and carbohydrate) may have a positive association with the risk of developing CRC [4,5]. Additionally, fruits and vegetables as sources of dietary fiber, folate, phytoestrogens, vitamin C, selenium, carotenoids, phenols, and flavonoids could protect against the development of CRC [6,7]. Antioxidants are reported to function by trapping free radicals and reactive oxygen molecules at the cellular level, thus acting as a protective mechanism against oxidative damage [6,7]. Free radicals in the body are generally produced during metabolic processes, such as those involving digestion. For example, iron found in red meat is reported to be a source of free radicals present in the body [8]. However, evidence suggesting red meat as a possible cause of colon cancer has been questioned by Santorelli *et al.* [9] in the CRC debate. Generally, in many households, meals high in fat are usually low in fruits, vegetables and fiber. Therefore, it is unclear if this increase risk in developing CRC is attributable to the high fat intake or the low fruit, vegetable and fiber intake. Free radical-induced lipid peroxidation has been implicated in malignant transformation [10]. The formation of lipid peroxidation products is normally prevented or scavenged by host antioxidants. Low levels of antioxidant nutrients in circulation have been associated with an increased risk of cancer [10].

One local Jordanian study published by Arafa *et al.* [11], showed in descriptive terms that traditional Jordanian foods are cooked with a high quantity of saturated fats and oils, and more importantly, anecdotal evidence exists that the fruits and vegetables component of the local diet is very low, while red meat and saturated fat components are quite high. More so, in the study by Arafa *et al.* [11], difference in the dietary macro- and micronutrient intake in the traditional diets were reported. The study results were descriptive in nature and did not investigate any association between diet and the risk of developing CRC. In addition, it is important to identify risk factors that could be modified to decrease CRC incidence among Jordanians. Based on current knowledge, the risk of developing CRC in the Jordanian population should be investigated in more detail. Accordingly, the aim of the present study is to investigate the association between macro-and micronutrient intake and colorectal cancer risk using data from a case-control study conducted in Jordan.

## 2. Materials and Methods

### 2.1. Study Population and Methods

The study sample consisted of 503 participants; with 232 diagnosed CRC cases and 271 controls (262 males and 241 females). Participants were enrolled in the study from January 2010 to December 2012. Participating subjects were patients diagnosed with CRC (cases) who were recruited from five Jordanian hospitals specializing in oncology diagnosis and treatment. The hospitals included King Hussein Cancer Center (KHCC), King Abdullah University Hospital, Prince Hamzeh Hospital, Jordan University Hospital, and Al-Basheer Hospital. The control group was recruited from hospital personnel, outpatients, visitors and was matched as closely as possible for age, gender, occupation and marital status. Control subjects were excluded if any first- or second degree relatives were diagnosed with CRC. The study protocol was approved by KHCC Institutional Review Board (IRB) Committee (09 KHCC 10; May 2009) and other hospitals gave their approval accordingly. Written informed consent was obtained from all subjects before their interview. The following inclusion criteria for controls were used: Jordanian nationals aged 18 years or older, ability to communicate clearly and verbally, free of any type of diagnosed cancer, diabetes mellitus, liver disease and rheumatoid arthritis. For inclusion in the diagnosed CRC cancer group, subjects must have received their diagnosis less than 1 year prior to the time of the first interview. The exclusion criteria for this group included those who were considered “critically ill”, such as an in-patient at any facility and those who were unable to communicate verbally and clearly.

### 2.2. Data Collection

Socio-demographic, health and dietary data were collected by trained research assistants using interview-based questionnaires. The socio-demographic data included age, marital status, household income, education (illiterate, primary and secondary, diploma and B.Sc., and postgraduate degrees), occupation and tobacco usage (current and previous smokers were categorized as smokers and those who never smoked were set as non-smokers). The comprehensive health data included the participant’s full medical history to confirm that only CRC diagnosed subjects and healthy disease free subjects were included. A validated Arabic quantitative Food Frequency Questionnaire (FFQ), adapted from the Diet History Questionnaire (DHQ I) of the National Cancer Institute of the United States of America [12], was used for dietary assessment. The FFQ questions sought to obtain information on the dietary history of study participants prior to CRC diagnosis, and to confirm the dietary habits of control participants. We selected a period of one year prior to the study inception date, to account for seasonal variation in food types. We noted a fixed dietary pattern for the period, with some participants suggesting this pattern existed for at least five years. A qualified dietitian asked participants, during face-to-face interviews, how frequently, on average, during the past year they had consumed one standard serving of specific food items in nine categories (<1/month, 2–3/month, 1–2/week, 3–4/week, 5–6/week, 1/day, 2–3/day, 4–5/day, or 6/day). Food lists in the modified FFQ questions were classified based on types of foods: 21 items of vegetables; 16 items of meat such as red meat (lamb and beef), chicken, fish, cold meat, and others; 21 items of fruits and juices; nine items of milk and dairy products; eight items of cereals; four items of beans; four items of soups and sauces; five items of drinks; nine items of snacks and sweets; and 14 items of herbs and spices [12]. Food models and standard measuring tools were used to help participants estimate portion size. Responses on frequency of consumption and serving size for each food item were converted into average daily intake. Data was collected from a total of 503 participants. However, the data from 86 participants was excluded due to incomplete response to required questions (*n* = 58); over-estimation of calorie intake (>5000 kcal for male and >4000 kcal for female) (*n* = 12); and under-estimation of calorie intake (<500 kcal for females and <800 kcal for males) (*n* = 16) [13]. Dietary intakes were analyzed using dietary analysis software (ESHA Food Processor SQL version 10.1.1; ESHA, Salem, OR, USA) with additional data on foods consumed in Jordan [14].

The 7-day Physical Activity Recall (PAR), developed by Sallis *et al.* [15] was used to measure physical activity level. 7-Day PAR is a structured interview that depends on participant’s recall of time spent engaging in physical activity over a seven day period. Our participants were asked specific and probing questions in order to obtain a complete history of their physical activities. They were asked to recall their physical activities for the previous year before their enrollment into the study. PAR covers different levels of physical activity and intensity such as aerobic exercise, work-related activities, gardening, walking, recreation, and leisure-time activities. The PAR interview focuses on collecting data on intensity, time or duration, and type of activity. The number of hours spent in different activity levels were obtained and converted into Metabolic Equivalents (METs). Average METs for walking = 3.3 METs, for moderate activity = 4.0 METs, for vigorous activity = 8.0 METs. The score expressed as MET-min/week was calculated as: (MET level × minutes of activity/day × days per week). Total Physical Activity MET-min/week is obtained by METs summation and categorized as inactive (below 600 MET-min/week), minimally active and Health Enhancing Physical Activity (HEPA) active. Minimally active category included subjects who reported a minimum of at least 600 MET-min/week. The category HEPA active included any subject who performed vigorous-intensity activity on at least 3 days a week and accumulated at least 1500 MET-min/week or who performed any combination of walking, moderate-intensity or vigorous intensity activities on 5 or more days achieving a minimum of at least 3000 MET-min/week [15].

Body weight was measured to the nearest 0.1 kg, with minimal clothing and without shoes, using a calibrated portable scale. Height was measured to the nearest 0.5 cm with participants in the full standing position without shoes using a calibrated portable measuring rod. Body mass index (BMI) was calculated as the ratio of weight in kilograms to the square of height in meters [16].

### 2.3. Statistical Analyses

Statistical analysis was performed with SPSS IBM-20 software. The significance level was set at *p* = 0.05. For descriptive statistics, mean ± standard deviation (SD) and percentages were used. T-tests evaluated the differences between cases and controls in continuous variables, and Chi-square was used to detect differences among categorical variables.

Because all nutrients were correlated with energy intake, variation due to energy intake and its associated measurement error was minimized by energy adjustment of the nutrients using the regression method [17]. This method of energy adjustment is computed from the residuals of the regression model with total energy intake as the independent variable and the nutrient as the dependent variable. Regression equation was used to calculate the expected mean of nutrient intake of the study population. Next, for each participant, the energy-adjusted intake was calculated by adding the expected mean nutrient intake of the study population to the residual derived from the regression analysis. Shapiro-Wilk test was used to assess the normality of the distributions of dietary intake variables. Non-normally distributed variables were log transformed [17].

Nutrient intakes were modeled using quartiles of distribution in the study population with quartile 1 being the lowest intake and quartile 4 the highest. Odds ratios (ORs) and 95% CIs (95% CIs) for CRC were calculated by using logistic regression models for quartiles of nutrient intakes, with the lowest quartile as the reference category. Confounders were selected based on known risk factors for CRC reported in the literature. Potential confounders were chosen based on previous studies [4,18] including the Cancer Prevention Study II [18]. Confounders included in data analysis included age, gender, BMI, physical activity (MET-min/week), family history (beyond the second degree) of CRC, household income, educational level, marital status and smoking. Trend tests were calculated using linear regression with nutrient intakes as continuous data.

## 3. Results

Table 1 shows participants’ age, anthropometric measurements, socio-demographic and health characteristics, stratified by gender. Average age for controls was 51.4 ± 10.9 years and 53.8 ± 12.2 years for cases. Significant differences were found between cases and controls in male height, and in female BMI. No significant differences were detected in employment, marital status, monthly income, smoking and physical activity levels between the CRC diagnosed and control participants. However, family history (beyond the second degree) of CRC and having other health problems in female participants was significantly higher in the CRC group compared to the controls.

The mean daily intakes of total energy, macronutrients, and micronutrients appear in Table 2. The CRC group reported significantly higher intakes of total energy, protein, fat, saturated fat and cholesterol (*p* < 0.05) compared to the control group. In addition, the CRC group had significantly higher intakes of folate, Iron, selenium as well as omega-3 (*p* < 0.05) when compared to the control group, and the control group had a higher percentage of calories from carbohydrate when compared to the CRC group (*p* < 0.05).

**Table 1:** Age, anthropometrics measurements and selected characteristics of the study participants.

Characteristics	Males (*n* = 193)			Females (*n* = 224)		
	Control (*n* = 113)	Case (*n* = 80)	*p*-value	Control (*n* = 135)	Case (*n* = 89)	*p*-value
Age years (mean ± SD)	55.2 ± 11.6	57.9 ± 12.1	0.110	48.1 ± 8.9	50.0 ± 11.0	0.161
Height cm (mean ± SD)	164.3 ± 9.6	169.5 ± 9.2	0.001	170.1 ± 40.5	166.8 ± 10.6	0.458
Weight kg (mean ± SD)	81.3 ± 16	81.1 ± 14.6	0.092	79.9 ± 14.9	77.5 ± 16.5	0.271
BMI kg/m^2^ (mean ± SD)	27.3 ± 4.8	27.8 ± 5.4	0.558	30.2 ± 6.0	27.6 ± 7.4	0.004
*Age Category n (%)*						
<40 years	9 (8)	10 (12.7)	0.397	22 (16.9)	14 (15.9)	0.095
40–49 years	25 (22.1)	12 (15.2)		57 (43.8)	32 (36.4)	
50–59 years	36 (31.9)	15 (19.0)		35 (26.9)	20 (22.7)	
≥60 years	43 (38.1)	42 (53.2)		16 (12.3)	22 (25.0)	
Total	113 (100.0)	79 (100.0)		130 (100.0)	88 (100.0)	
*Employed (%)*						
Yes	63 (55.8)	30 (37.5)	0.081	26 (19.3)	21 (23.6)	0.510
No	50 (44.2)	50 (62.5)		109 (80.7)	68 (76.4)	
Total	113 (100.0)	80 (100.0)		133 (100.0)	89 (100.0)	
*Marital status n (%)*						
Married	106 (93.8)	76 (95)	0.695	113 (83.7)	74 (83.2)	0.364
Single	4 (3.5)	2 (2.6)		10 (7.4)	2 (2.2)	
Divorced	-	1 (1.3)		1 (0.7)	1 (1.1)	
Widowed	3 (2.7)	1 (1.3)		11 (8.1)	12 (13.5)	
Total	113 (100.0)	80 (100.0)		135 (100.0)	89 (100.0)	
*Family history (beyond the second degree) of CRC n (%)*						
Yes	45 (40.2)	27 (33.8)	0.475	45 (34.1)	42 (47.7)	0.016
No	67 (59.8)	53 (66.3)		87 (65.9)	46 (52.3)	
Total	112 (100.0)	80 (100.0)		132 (100.0)	88 (100.0)	
*Other health problem n (%) (excluding diabetes, liver disease, rheumatoid arthritis)*						
Yes	45 (40.2)	45 (57.0)	0.367	47 (34.8)	40 (44.9)	0.043
No	67 (59.8)	34 (43.0)		88 (65.2)	48 (53.9)	
Total	112 (100.0)	79 (100.0)		135 (100.0)	88 (100.0)	
*Tobacco use n (%)*						
Yes	36 (34.0)	17 (23.0)	0.113	4 (3.0)	7 (8.0)	0.093
No	70 (66.0)	57 (77.0)		131 (97.0)	81 (92.0)	
Total	106 (100.0)	74 (100.0)		135 (100.0)	88 (100.0)	
*Household income n (%)*						
<300 USA $	10 (8.8)	6 (7.4)	0.663	15 (11.0)	5 (5.6)	0.562
300–750 USA $	27 (23.9)	19 (23.8)		41 (30.4)	24 (27.0)	
>750 USA $	65 (57.6)	36 (45.0)		44 (32.6)	24 (27.0)	
Unknown	11 (9.7)	19 (23.8)		35 (26.0)	36 (40.4)	
Total	113 (100.0)	80 (100.0)		135 (100.0)	89 (100.0)	
*Physical activity levels n (%)*						
Inactive *	30 (27.0)	27 (33.8)	0.469	10 (9.1)	16 (18.0)	0.183
Minimally Active ^†^	40 (36.0)	25 (31.3)		33 (30.0)	23 (25.8)	
HEPA active ^‡‡^	41 (36.9)	28 (35.0)		67 (60.9)	50 (56.2)	
Total	111 (100.0)	80 (100.0)		110 (100.0)	89 (100.0)	

BMI: Body Mass Index; * Inactive: not fitting in “Minimally Active” or “HEPA active”; Significance is at *p* ≤ 0.05. ^†^ Minimally Active: at least 600 MET per week; ^‡‡^ Health Enhancing Physical Activity: HEPA active: more than 3000 MET per week.

**Table 2:** Mean intake per day ± SD of nutrients for the study participants.

Nutrient	Control	Case	Difference (Case − Control)	*p*-value *
Energy (kcal)	3476.0 ± 1172.9	3719.4 ± 1018.1	243.4	0.029
Protein (g)	109.6 ± 42.5	120.9 ± 52.4	11.3	0.016
Protein %	12.5 ± 2.3	13 ± 3.7	0.4	NS
Carbohydrate (g)	593.9 ± 203.1	608.1 ± 164.6	14.2	NS
Carbohydrate %	68.9 ± 8.6	66.1 ± 8.4	−2.8	0.001
Fiber (g)	48.4 ± 23.3	48.1 ± 21.3	−0.3	NS
Soluble Fiber (g)	6.0 ± 4.5	5.4 ± 3.5	−0.7	NS
Insoluble Fiber (g)	14.7 ± 10.8	13.1 ± 9.1	−1.5	NS
Fat (g)	80.2 ± 39.1	93.8 ± 41.3	13.6	0.001
Fat %	20.4 ± 6.6	22.1 ± 6.5	1.7	0.009
Saturated Fat (g)	29.8 ±16.3	35.4 ± 17.1	5.6	0.001
Saturated Fat %	7.6 ± 3.2	8.4 ± 3.2	0.8	0.009
Cholesterol (mg)	340.2 ± 229.0	409.3 ± 273.7	69.1	0.005
Vitamin A (RE)	1116.5 ± 850.5	1199.8 ± 871.6	83.3	NS
Beta-carotene (μg)	6478.7 ± 5259.1	6822.6 ± 556.3	343.9	NS
Vitamin B_12_ (μg)	3.9 ± 3.5	4.5 ± 4.3	0.6	NS
Vitamin C (mg)	239.2 ± 173.1	259.7 ± 207.4	20.5	NS
Vitamin D (mg)	0.8 ± 0.7	0.9 ± 0.7	0.1	NS
Vitamin E (α-Tocopherol) (mg)	6.4 ± 3.9	6.7 ± 4.1	0.2	NS
Folate (mcg)	461.3 ± 217.2	506.3 ± 186.6	44.9	0.029
Vitamin K (μg)	193.3 ± 203.8	197.5 ± 174.3	4.1	NS
Calcium (mg)	1171.1 ± 526.9	1230.5 ± 459.2	59.4	NS
Iron (mg)	25.2 ± 9.9	27.4 ± 10.1	2.3	0.022
Sodium (mg)	4796.2 ± 2837.5	5112.0 ± 2218.6	315.8	NS
Selenium (μg)	109.3 ± 53.3	120.1 ± 48.9	10.9	0.033
Phosphate (mg)	1334.4 ± 561.7	1416.6 ± 619.5	82.2	NS
Omega-3 (mg)	0.6 ± 0.4	0.7 ± 0.5	0.1	0.014
Caffeine (mg)	3036.6 ± 2829.8	2980.7 ± 3041.8	−55.9	NS

^*^ Significance is at *p* < 0.05.

Table 3 shows the ORs and corresponding 95% CI of the CRC group by intake quartile of associated macronutrients. After adjusting for potential confounders, increasing intakes (in the highest *versus* the lowest quartile of intake) of total energy (OR = 2.60, 95% CI: 1.22–5.56, *p*-trend = 0.030), and protein (OR = 3.62, 95% CI: 1.63–8.04, *p*-trend = 0.002) were significantly associated with CRC. A significant positive trend in risk was found for carbohydrate (*p* = 0.043), but none of the quartiles are different from the reference category. The odds ratios for quartiles of fat intake as g/day were not calculated due to distribution issues (the bottom quartile had only 1 participant and the top quartile only 2 control participants). As noted in Table 3, saturated fat and cholesterol intakes show significant direct associations with CRC risk (OR = 5.23, 95% CI: 2.33–11.76 and OR = 2.48, 95% CI: 1.18–5.21, respectively) in the highest *versus* the lowest quartile of intake, and the trend tests were also significant. No association for intake of total fiber with CRC was detected, (*p*-trend = 0.979, Table 3). However, the upper quartile of insoluble fiber was found to be protective against CRC (OR = 0.42, 95% CI: 0.19–0.91) but the trend test was not significant (*p*-trend = 0.162).

**Table 3:** Adjusted ORs ^a^ and CIs of CRC risk by macronutrient intake quartiles.

Nutrient	Adjusted				
	Q1	Q2	Q3	Q4	*p*-Trend
*Energy (Kcal)*					0.030
No. of Cases (169)/Controls (248)	32/72	45/60	44/60	48/56	
OR	1	1.51	1.83	2.60 *	
95% CI	-	0.70–3.27	0.85–3.93	1.22–5.56	
*Protein (g)*					0.002
No. of Cases (169)/Controls (248)	30/74	37/67	46/58	56/48	
OR	1	1.66	1.74	3.62 *	
95% CI	-	0.74–3.69	0.78–3.90	1.63–8.04	
*Carbohydrate (g)*					0.043
No. of Cases (169)/Controls (248)	36/68	37/67	43/62	53/51	
OR	1	0.77	1.24	1.41 *	
95% CI	-	0.36–1.64	0.58–2.64	0.68–2.99	
*Fiber (g)*					0.979
No. of Cases (169)/Controls (248)	46/58	49/56	33/71	41/63	
OR	1	1.29	0.48	0.57	
95% CI	-	0.62–2.69	0.22–1.04	0.27–1.21	
*Soluble Fiber (g)*					0.551
No. of Cases (169)/Controls (248)	43/61	53/52	39/65	34/70	
OR	1	1.86	0.85	0.58	
95% CI	-	0.84–4.15	0.39–1.84	0.26–1.27	
*Insoluble Fiber (g)*					0.162
No. of Cases (169)/Controls (248)	46/58	51/54	40/64	32/72	
OR	1	1.04	0.66	0.42	
95% CI		0.49–2.22	0.30–1.41	0.19–0.91	
*Fat ^b^ (g)*					
No. of Cases (169)/Controls (248)	0/104	4/100	63/42	102/2	
OR	-	-	-	-	
95% CI	-	-	-	-	
*Saturated Fat (g)*					0.009
No. of Cases (169)/Controls (248)	28/76	36/69	52/52	53/51	
OR	1	2.23	3.61	5.23 *	
95% CI	-	1.00–4.98	1.68–7.77	2.33–11.76	
*Cholesterol (mg)*					0.027
No. of Cases (169)/Controls (248)	33/71	32/72	49/56	55/49	
OR	1	0.94	1.84	2.48 *	
95% CI	-	0.43–2.05	0.87–3.91	1.18–5.21	

^a^ Adjusted for total energy intake normality of the distributions of dietary intake variables was assessed by the Shapiro-Wilk test. Non-normally distributed variables were log transformed. Other potential confounders included age, gender, BMI, physical activity (METs/week), family history (beyond the second degree) of CRC, education attainment, household income, marital status and tobacco use; ^b^ Odds ratios were also calculated for percentage of energy from fat using the following categories: 1 (≤20% of energy), 2 (20%–35% of energy), 3 (≥35% of energy). The ORs for category 2 and category 3 relative to category 1 were 1.80 (95% CI: 1.07–3.04), and 2.10 (95% CI: 0.38–11.70), respectively, with *p*-trend = 0.009. * Significant different from reference category, *p* ≤ 0.05.

Vitamin E intake showed significant protective effect against CRC with OR = 0.02 and 95% CI: 0.0003–0.011, (Table 4). Neither quartile analysis nor the trend test was significant for vitamins A, C, B_12_, D, K, and folate, beta-carotene, phosphate, and omega-3. Calcium showed a significant risk in the top two quartiles.

**Table 4:** Adjusted ORs ^a^ and CIs of CRC risk by micronutrient intake quartiles.

Nutrient	Adjusted				
	Q1	Q2	Q3	Q4	*p*-Trend
*Vitamin A (RAE)*					0.769
No. of Cases (169)/Controls (248)	42/62	38/67	43/61	46/58	
OR	1	0.90	0.87	0.77	
95% CI	-	0.43–1.89	0.43–1.77	0.37–1.58	
*Beta-carotene (μg)*					0.575
No. of Cases (169)/Controls (248)	42/62	39/66	63/41	47/57	
OR	1	0.78	0.78	0.71	
95% CI	-	0.38–1.59	0.38–1.63	0.33–1.55	
*Vitamin B_12_ (μg)*					0.493
No. of Cases (169)/Controls (248)	45/59	31/73	40/65	53/51	
OR	1	0.38	0.66	1.07	
95% CI	-	0.17–0.83	0.32–1.35	0.52–2.222	
*Vitamin C (mg)*					0.359
No. of Cases (169)/Controls (248)	41/63	40/65	40/64	48/56	
OR	1	0.81	0.63	0.89	
95% CI	-	0.40–1.62	0.29–1.34	0.42–1.89	
*Vitamin D (mg)*					0.163
No. of Cases (169)/Controls (248)	39/68	43/59	40/64	47/57	
OR	1	1.07	1.33	1.47	
95% CI	-	0.50–2.31	0.64–2.76	0.70–3.08	
*Vitamin E (α-Tocopherol) (mg)*					0.001
No. of Cases (169)/Controls (248)	95/9	62/43	12/92	-/104	
OR	1	0.05	0.02	-	
95% CI	-	0.01–0.23	0.0003–0.011	-	
*Folate (μg)*					0.057
No. of Cases (169)/Controls (248)	33/71	41/64	46/58	49/55	
OR ^a^	1	1.32	1.24	1.14	
95% CI	-	0.62–2.84	0.58–2.66	0.54–2.42	
*Vitamin K (μg)*					0.612
No. of Cases (169)/Controls (248)	37/67	41/63	48/57	43/61	
OR	1	0.95	1.12	0.95	
95% CI	-	0.68–2.13	0.88–2.70	0.74–2.29	
*Calcium (mg)*					0.146
No. of Cases (169)/Controls (248)	29/75	40/65	55/49	45/59	
OR	1	1.80	3.92	2.39	
95% CI	-	0.82–3.96	1.81–8.50	1.04–5.52	

^a^ Adjusted for total energy intake normality of the distributions of dietary intake variables was assessed by the Shapiro-Wilk test. Non-normally distributed variables were log transformed. Other potential confounders included age, gender, BMI, physical activity (METs/week), family history (beyond the second degree) of CRC, education attainment, household income, marital status and tobacco use.

## 4. Discussion

The results from of the present study provide further evidence for an association between CRC risk and diet. Generally, the results of this case-control study on CRC risk illustrate a relationship between macro- and micronutrients intake and this type of cancer among Jordanians.

As BMI was obtained at the time of interview for both patients and controls, the association between obesity and CRC in this study could not be evaluated. The lower BMI in cases may reflect the effect of chemotherapy and other therapies which cancer patients were exposed to before the interview time.

Our study revealed a direct association between total energy intake and the risk of developing CRC, as supported by several other studies [4,19]. Caloric restriction was found to reduce cancer incidence in rodents and colorectal cell proliferation in humans [20]. The potential mechanism could be through insulin growth factor-1 (IGF-1), where increasing energy could be responsible for glycemic overload and a compensatory increase of serum insulin and related IGF-1, a promoter of tumor cell growth *in vitro* [21,22]. Elevated circulating insulin and IGF level may increase CRC risk, possibly by decreasing IGF-binding proteins (IGFBP-1) and increasing the bioactivity of IGF-I [23,24]. Insulin may increase the circulating IGF-1/IGFBP-3 ratio by increasing hepatic growth hormone sensitivity which could be implicated in increasing the risk for CRC [23,24].

High carbohydrate intake may increase glycemic load, insulin levels, and IGF-1 [20,21]. A significant trend for higher intake of carbohydrate was detected among cases compared to controls. This observation is consistent with some studies [25,26,27] but not all [4,28]. Borugian *et al.* [26] reported a significant positive association between carbohydrate intake and risk of CRC in both men (OR = 1.7; 95% CI: 1.1–2.7) and women (OR = 2.7; 95% CI: 1.5–4.8) among Chinese in North America. While, Franceschi *et al.* [27] found a direct association between dietary glycemic load and CRC risk, with OR of 1.7 (95% CI: 1.5–2.2).

The effect of fiber on CRC incidence is inconsistent; some studies report a significant inverse association between total fiber intake and CRC risk [4,29,30,31], whereas other studies found no association between fiber intake and CRC incidence [27,32,33,34]. Although our results showed no association for the intake of total fiber with CRC, a significant protective effect of insoluble fiber on the risk of CRC development at the highest quartile has been detected [32]. A prospective cohort study of women in the United States, found that total fiber was not associated with CRC risk, with relative risk (RR) for the highest relative to lowest quintile of 0.75 (95% CI: 0.48–1.17, *p*-trend = 0.12). In the other two mentioned studies, significant associations in age-adjusted models disappeared after adjustment for other risk factors. In the Pooling Project analysis including data from 13 cohort studies the report showed statistically significant inverse associations for colorectal cancer in the age adjusted models (Quintile 5 *vs.* Quintile 1, RR 0.84, 95% CI: 0.77–0.92), but not after multivariable adjustment (Quintile 5 *vs.* Quintile 1, RR 0.94, 95% CI: 0.86–1.03) [33]. Similarly, in an NIH-AARP analysis the statistically significant inverse association in the age adjusted model (Quintile 5 *vs.* Quintile 1, HR 0.73, 95% CI: 0.65–0.82) disappeared after multivariable adjustment (Quintile 5 *vs.* Quintile 1, RR 0.99, 95% CI: 0.85–1.15) [34].

Similar to other studies [4,12,26,35], our study results show that total fat, saturated fat and cholesterol have a significant direct effect on CRC risk. The total consumption of fat was much higher in our CRC participants than controls, with only one control in the first quartile. Our observation on fat, saturated fat and cholesterol is in agreement with the report of Arafa *et al.* [11] who reported the daily intake from saturated, mono and polyunsaturated fats and cholesterol is significantly higher among CRC diagnosed subjects as compared to controls, (*p* < 0.05). The proposed mechanism for fat involvement in colorectal carcinogenesis appears to be complex. However, Endo *et al.* [35], showed that the molecular mechanisms underlying the promotion of colorectal carcinogenesis by a high-fat diet (HFD) is through its effect on the role of the insulin-signal pathway and the c-Jun *N*-terminal kinase (JNK) pathway, which was reported to play a crucial role in insulin resistance during colorectal carcinogenesis in the presence of hyperinsulinaemia induced by a HFD. They found that colonic cell proliferation was promoted via the JNK pathway in the presence of a HFD providing an explanation of the effect of dietary fat intake on colon carcinogenesis through the JNK pathway [35].

In our study, protein intake was found to have a significant direct association with CRC. Arafa *et al.* [11] indicated that the consumption of protein among CRC diagnosed patients was higher than intake in a control group, and they speculated that this may be associated with a higher risk for development of CRC. In contrast, one other study by Sun *et al.* [5], reported an inverse association for intake of protein (OR: 0. 85, 95% CI: 0.69–1.00, *p*-trend = 0.002, 4th *versus* 1st quartile). However, Egeberg *et al.* [36] reported a significant association between specific red meat subtypes intake and the risk of developing colon and rectal cancers. They found that consuming lamb meat was significantly related to risk of developing colon cancer, while consuming pork meat was significantly related to the risk of developing rectal cancer [36]. No associations were found between intake of red meat, processed meat, fish, or poultry and risk for colon cancer or rectal cancer [36]. Fifty percent of participants consumed red meat more than 1–2 times per week (results not shown) in serving size ranges from 90–120 gm. However, the majority (80%) consumed poultry more than 3–4 times weekly. This may partially explain why protein intake in this study was a CRC risk factor rather than protective. In a previous report, Tayyem *et al* [37], we reported that the Jordanian population consumes more animal proteins than plant proteins [37]. In fact, meat intake increased from 7.68 kg/year per-capita in 1961 to 35.85 kg/year per-capita in 2005 [37].

In our study, vitamin E was found to have a significant inverse association with the risk of CRC development. The remarkable effect of vitamin E consumption on protecting against CRC could be attributed to the comparative ratio of 95 case/9 control participants at the lowest quartile compared to 12 case/92 control participants at the 3rd quartile of vitamin E consumption. These results are in agreement with other studies [31,38,39,40,41]. A study of Satia-Abouta *et al.* [29], that had been conducted on African Americans, revealed that vitamin E intake was strongly and inversely associated with a 70% reduced risk for colon cancer (OR 0.3; 95% CI (0.1–0.6)). This trend of association was not demonstrated in the same study in Whites (OR 1.0; 95% CI (0.6–1.6)). This could be attributed to ethnic differences [29]. Perhaps, this may be due to genetic makeup or the influence of genes in the metabolic processes. This effect may arise from vitamin E activity as an antioxidant against free radicals and reactive oxygen molecules [39].

The association between folate intake and CRC disease state is being debated and no significant association between folate and CRC risk has been reported [26,42,43,44,45], similar to results obtained in our study. However, recent research indicates that folate may have a role in the metabolism of colon carcinogenesis, perhaps by increasing 5, 10-methylenetetrahydrofolate levels for DNA synthesis [44,45].

Iron (Fe) intake was found to have a positive weak association with CRC risk [24]. Our results are consistent with those from other studies [46,47,48]. In Larsson *et al.* [46] study, the RR of colon cancer, comparing extreme categories of heme iron intake, was 2.29 (95% CI: 1.25–4.21) among women who consumed at least 20 g/week of alcohol, and 1.05 (95% CI: 0.74–1.48) among women who consumed less than 20 g/week of alcohol. The molecular mechanisms of iron carcinogenesis may be explained by the actions of auto-oxidation of iron involving only Fe^2+^ + O_2_ in oxidant formation in biological systems and its pH dependency, activation of oxidative responsive transcription factors and pro-inflammatory cytokines, and iron-induced hypoxia signaling [47,48].

In our study, the intake of selenium was found to have a significant trend for direct association with CRC risk. Other studies reported the presence of an inverse association between selenium intake and plasma levels with CRC risk [49,50,51]. Our results may be explained by the finding of Whanger [51] who conducted a study on the form of selenium compounds with CRC risk. His results suggested that Selenomethionine (Semet), the major seleno-compound in cereal grains and enriched yeast, may be the effective form of selenium against CRC. However, Whanger [51] found that Se-methylselenocysteine (SeMCYS), the major seleno-compound in Se-accumulator plants and some plants of economic importance such as garlic and broccoli, may be ineffective against CRC and only protect against mammary tumors. Our results suggest that the effective form of selenium that could protect against CRC disease may have been limited in our participants’ diets.

Some studies have shown an association between high sodium intake and CRC development [52,53,54], and Zhivotovskiy *et al.* [54] have reported that the risk of CRC increased almost 3.5-fold as the dietary intake of salt increased with *p*-trend of 0.008.The results of our study is in agreement with these reports. One potential explanation for this is the presence of chemical carcinogens such as *N*-nitroso compounds in salted foods such as processed meats, dairy products and canned foods, which can be formed by the reaction of sodium nitrate or sodium nitrite in the curing process or in the body, or heterocyclic amines, which have been detected in fish or meat cooked in high temperatures, such as grilling, which is commonly used for grilled meat [54].

Regarding caffeine intake, our results are in agreement with other studies. Caffeine has been shown to have a negative association with CRC risk [55,56]. It inhibits colon cancer cell growth, by acting as antioxidant and effectively scavenging hydroxyl radicals (·OH) [55]. Additionally, caffeine can decrease insulin sensitivity, possibly as a result of elevated plasma epinephrine levels [56].

## 5. Study Limitation

In a study of this type we rely greatly on the ability and memory recall of participants to accurately and carefully provide information from a period when it was not necessarily important to remember the details of long digested meals, or physical activities that were undertaken. It is understandable that some individuals may have a greater recall than others, and that biases may exist in the minds of those being interviewed, and indeed, by the interviewer. Traumatic events such as the diagnosis of a life threatening disease condition may ultimately have a very significant role in the memory recall of some participants. Because of the obvious limitations placed on the recall of memory, we are using the only means currently available to us, the FFQ, which although prone to errors, is nevertheless an accepted and validated form used in many research studies.

We did not take into account the possible effects of cooking on the bioavailability of the various nutrients, and although we attempted to control for a range of potential confounders, we did not measure alcohol use (culturally discouraged). Nor did we consider the use of food dietary supplements; however, we are aware that the use of dietary supplements is not common or widespread. Finally, the one year dietary recall time frame may not be sufficient to determine an association with a disease state that may take years to develop; nevertheless, we see this study as a pointer to the need for further long term studies involving journal and diary entries of nutritional intakes along with physical activities for designated period of time, whether it be five to fifteen years.

A major strength of our study is the validated and detailed FFQ used to collect dietary data from our study population. Even though dietary data were collected at only one time, the FFQ has been reported to be an adequate instrument for measuring macro- and micronutrient intake [12]. Confirmative studies should verify and extend the presented data on Jordanian dietary habits in order to establish recommendations for people in Jordan to decrease colorectal cancer incidence.

## 6. Conclusions

This study, conducted in a Jordanian population group provides additional evidence that diets containing high energy, protein, total fat, saturated fat, cholesterol, and sodium intakes may increase the risk of CRC development, whereas high intakes of insoluble fiber, vitamin E, and caffeine may decrease the risk of these diseases. These results suggest that dietary changes could help to reduce the incidences of CRC in the Jordanian population.

## Abbreviation

CRC: Colorectal cancer
FFQ: Food Frequency Questionnaire
DHQ I: Diet History Questionnaire I
PAR: 7-day Physical Activity Recall
HEPA: Health Enhancing Physical Activity
METs: Metabolic Equivalents
BMI: Body mass index
